# The New Lunacy Act for the "Regulation of the Care and Treatment of Lunatics"

**Published:** 1853-10-01

**Authors:** 


### THE NEW LUNACY ACT FOR THE "REGULATION OF THE CARE AND TREATMENT OF LUNATICS."

### 16 and 17 Vict., cap. 96.

The commissioners in lunacy have issued a circular, addressed to the proprietors and superintendents of private and public lunatic asylums, requiring them, in accordance with its 37th section, to have a Queen's printer's copy of this Act, 16 and 17 Victoria, cap. 96, entitled, "An Act to amend an Act passed in the ninth year of her Majesty, 'For the Regulation of the Care and Treatment of Lunatics,'" bound up in the "Visitor's-book," along with the former Act, 8 and 9 Victoria, cap. 100. We propose, therefore, now to enumerate the various alterations which have been made in the different sections and clauses of this Bill, with the view of pointing out the new provisions and regulations which will come into operation on the 1st of November next. We shall, for convenience, follow the same sectional arrangement which we adopted in the analysis of the Act 8 and 9 Victoria, so that our present article, which will refer only to the new points in the Bill before us, may not be an unacceptable sequel to the little volume which we published a few years ago.*

*Licences. Two or more separate houses may be included in one Licence.*—Section 1, 16 and 17 Vict., cap. 96, repeals the 25th section 8 and 9 Vict. cap. 100, which enacted that no licence should include more than one house, and defines with precision what may be included in one licence. "Any one licence to be granted for the reception of lunatics may, in the discretion of the commissioners or justices granting such licence, include two or more houses belonging to one proprietor, or to two or more joint proprietors, provided that no one of such houses be separated from the other or others of them otherwise than by land in the same occupation, and by a road, or by either of such modes; and all houses, buildings, and lands, intended to be included in any licence, shall be specified, delineated, and described, by section 24 of the said recited Act." That is to say, the same plan of the premises, accompanied with an account of the applicant's position and qualifications, will be required, as was before required by sect. 24 of the Act 8 and 9 Vict., cap. 100.

*Order of Reception.*—Under the new act, no person, whether a pauper or a private patient, is, as before, to be boarded or lodged in any licensed house, without an order of reception and two medical certificates being duly filled up and signed; but the form of the order of reception, and the forms also of the medical certificates, are altered in the following respects—in addition to the particulars already required, a statement is to be made in the order of reception as to "When and where previously under care and treatment"—"Duration of existing attack" (instead of "length of time insane")—"Supposed cause"—and the question "Whether suicidal or dangerous to others," is divided into two separate paragraphs "Whether suicidal," and "Whether dangerous to others." It is also required, in the order for the reception of a pauper patient, that "The parish or union to which the lunatic is chargeable," shall be stated; and also "The name and christian name, and place of abode, of the nearest known relative of the patient, and the degree of relationship, if known."

*Medical Certificates.*—Two medical certificates, as before, will be required for the admission of a private patient, and one for the admission of a pauper patient. They differ, however, from the previous forms in this respect, that

* An Act (8 & 9 Vict., c. 100) for the "Regulation of the Care and Treatment of Lunatics, with Explanatory Notes and Comments," &c. &c. Edited by Forbes Winslow, M.D. London: Benning & Co.; Henry Renshaw, 1845.

under two separate heads must be specified "Facts indicating insanity observed by the medical practitioner who signs the certificate;" and "Other facts (if any) indicating insanity, are to be enumerated, which may have been communicated to him." Admitted into the asylum, the usual "notice of admission" to be sent to the commissioners in lunacy, and to the clerk of the peace in the case of pauper lunatics; but instead of two forms for the notice of admission—one for a private, another for a pauper patient—the same form is prescribed, with a blank for the insertion of the word private or the word pauper, as the case may be. The same statement from the medical proprietor or superintendent as is at present, is required to be filled up, and transmitted as before with the notice of admission. Patients may still be admitted into unlicensed houses upon *one* medical certificate, but *two* additional certificates must be obtained within three days after the admission of the patient into the asylum or hospital. The former act required only *one* additional certificate. A very important amendment, connected with medical certificates, is introduced by section 11, which provides that the orders of reception and the medical certificates may, if informal, after the reception of a patient, be returned for correction, under the sanction of one or more of the commissioners. "If, after the reception of any lunatic, it appear that the order or the medical certificate, or (if more than one) both or either of the medical certificates upon which he was received, is or are in any respect incorrect or defective, such order and medical certificate, or certificates, may be amended by the person signing the same at any time within fourteen days next after the reception of such lunatic; provided, nevertheless, that no such amendment shall have any force or effect, unless the same shall receive the sanction of one or more of the commissioners."

*Residence of persons in asylums without medical certificates.*—Under this clause, which is one of the most important alterations in the Act, *a discharged patient may continue to reside in an asylum, or a relative or friend of a patient may, with the assent of the visitors or commissioners, be received into the asylum, and may reside with an insane patient.* "It shall be lawful (sect. 6) for the proprietor or superintendent of any licensed house, with the previous assent in writing of two of the commissioners, such assent not to be given until after such commissioners have, by personal examination of the patient, satisfied themselves of his desire to remain, to entertain and keep in such house as a boarder any person who may have been discharged as patient from such house for such time after such discharge as he may desire to remain, not exceeding the time specified in such assent; and also, for the benefit of any patient in such house, and with the previous assent in writing of two of the commissioners, to receive and accommodate as a boarder therein, for a time to be specified in the assent, any relative or friend of such patient, and any two of the commissioners may, from time to time, by any writing under their hands, extend or revoke any such assent as aforesaid; and every such patient so retained after discharge, and every such relative or friend so accommodated, shall, if required, be produced to the commissioners and visitors respectively at their respective visits." In the case of paupers, such persons are to be included in the number of patients returned as being inmates of such house.

*Single patients.*—The amended act for the protection of single patients provides (sect. 8) that the same order of admission and medical certificates shall be required on the reception, or taking charge of any single person as a lunatic, as would be required in a licensed house; and furthermore, that the commissioners (sect. 14) may permit medical visitation of any single patient less frequently than once a fortnight. If, however, the patient be under the care of a medical man, he is to make an entry once a fortnight as to the patient's health. Visitors of licensed houses may also visit these single patients at the request of the commissioners.

By sect. 15, single patients in unlicensed houses are to be placed as nearly as possible upon the same footing as those who are confined in regularly licensed asylums. The sections also of the Act 8 and 9 Vict., cap. 100, prescribing the forms which are to be observed for the discharge or removal of patients in licensed houses (sects. 72 and 73), are to be extended to single patients in unlicensed houses.

Furthermore, sect. 22 provides that notice of change of residence, or removal of single patients from one residence to another, must be transmitted to the commissioners; and persons having the charge of a single patient must "obtain the consent of two of the commissioners to take or send such patient under proper control to any specified place or places, for any definite time, for the benefit of his health."

*Notice to be given to friends on the recovery of a patient.*—The medical superintendent or proprietor of every licensed house is hereafter to take the initiative in obtaining the discharge of such patients as may have recovered, inasmuch as with a view to their removal. It is provided that he shall notify their recovery to their friends; and in case such patients be not discharged or removed within fourteen days, a notice to that effect must be transmitted to the commissioners or visitors. (Sect. XIX.)

*Notice in case of death to be sent to the coroner of the county or borough.*—This very important addition to the above section appears to have been suggested in committee, as from the copy of the Bill before us—brought from Lords, 11th May, 1853, and printed as ordered by the House of Commons, 12th May, 1853, this clause does not appear. It was, therefore, one of the amendments introduced in committee. After the word apothecary, section 19, therefore, proceeds as follows:—"*And in case of the death of any patient in any hospital or licensed house, a statement setting forth the time and cause of the death, and the duration of the disease of which such patient died, shall be prepared and signed by the medical person or persons who attended the patient during the illness which terminated in death; and such statement shall be entered in the 'Case Book,' and a copy of such statement, certified by the superintendent or proprietor, shall, within two days of the date of the death, be transmitted to the CORONER of the County or Borough; and in case such Coroner, after receiving such statement, shall think that any reasonable suspicion attends the cause and circumstances of the death of such patient, he shall summon a jury to inquire into the cause of such death.*" We remember the late Dr. Millingen, after leaving Hanwell Asylum, where he had been the resident medical superintendent, writing in a somewhat irate tone against all lunatic asylums, designated them Bastilles, and proposed that they should be placed so far upon a level with gaols, that a "coroner's inquest should be held upon all lunatics who died out of the infirmary." This suggestion we certainly never expected to see adopted, and we apprehend that there will be considerable difficulty in the coroner of any county founding any opinion from the duration of the illness of the lunatic and thereupon determining the necessity of an inquest. Insanity, complicated with structural disease of the vital organs—the brain—the heart—the lungs, often terminates in death, preceded by only a very few days', or it may be hours', illness. The short duration of the illness ought not to be considered any criterion of maltreatment, neither ought it to suggest any suspicion that would warrant the holding of an inquest. The coroners of counties, by this section, have a new duty imposed upon them which few of them could have anticipated. It is worthy of observation that this clause *only applies to patients dying "in any hospital or licensed house," and does not apply to single patients dying in unlicensed houses;* but we shall reserve our observations upon this subject for a future occasion.

*Notice of the dismissal of attendants.*—By section 26, the superintendent or proprietor of every registered hospital or licensed house, within one week after the dismissal for misconduct of any nurse or attendant employed in such hospital or house, is to transmit to the commissioners, by the post, information in writing under his hand, of such dismissal, and of the cause thereof; and every superintendent or proprietor neglecting to transmit such information to the commissioners within the period aforesaid shall, for every such offence, forfeit any sum not exceeding ten pounds.

*Private committee of commissioners superseded.*—Under the former Act (8 and 9 Vict. cap. 100, sect. 89), the permanent chairman, for the time being, of the commissioners, and two other commissioners—one being a physician or surgeon, and the other a barrister—constituted what was called the Private "Committee," the duties of which were very onerous, and included, particularly, the difficult supervision of single lunatics in unlicensed houses. In several of the commissioners' late reports to the Lord Chancellor, they have pointed out how difficult it was for so limited a number of them to discharge the multifarious official duties which devolved upon them. Hence, section 27 of the new Act provides that "section 89 of the said recited Act, constituting from among the commissioners a private committee for the purposes in the said Act mentioned, shall be repealed, and all the powers vested in, and all the provisions of the said Act applicable to, the said private committee, or one or two members thereof, shall be vested in, and be applicable to, the commissioners, or one commissioner, or two commissioners (as the case may require), as if, where in the said Act the said private committee, or one member, or two members thereof (as the case may be), is or are mentioned or referred to, the commissioners, or one commissioner, or two commissioners (as the case may require), had been mentioned or referred to, instead thereof."

*Provision for the visitation of workhouses.*—By section 3 of the former Act, two or more of the commissioners—one being, at least, a physician, and one a barrister—were required once, or oftener, every year to visit every parish or union workhouse in which any lunatic, or alleged lunatic, was reported to be, and to inquire whether all the provisions of the law as to lunatics had, in such cases, been carried out. Here, again, very onerous duties devolved upon the commissioners, taxing their time beyond the possibility of achieving all the Act contemplated. This section of the Act has been repealed; and it is now, by section 28, provided that "any *one* or *more* of the commissioners," may discharge the duties "formerly delegated to the private committee."

*Commissioners in special cases may employ persons to make inquiries and report thereon.*—The duties of the commissioners will be facilitated by this section, which authorizes them to employ persons not officially connected with their Board to visit, upon special occasions, any lunatic asylum, and "report to them upon the mental and bodily state and condition of any lunatic, or alleged lunatic, in any asylum, hospital, or licensed house, or of any pauper lunatic in a workhouse or elsewhere, or of any lunatic, or alleged lunatic, under the care or charge of any person as a single patient, and to inquire into, and report upon, any matters into which the commissioners are authorized to inquire; and every such person shall, for the special purposes mentioned in such order, have all the powers of a commissioner; and the commissioners may allow to every such person a reasonable sum for his services and expenses, such sum to be paid in manner provided by the said recited Act with regard to expenses incurred by or under the authority of the commissioners in proceedings thereunder; but this enactment shall not be taken to exonerate the commissioners from the performance of any duty by law imposed on them."

*Commissioners to submit the regulations of registered hospitals to the Secretary of State.*—This section applies to the registered hospitals of Bethlehem, St. Luke's, Guy's, and other hospitals for the reception of lunatics, fourteen in number, by the last report of the commissioners. This section has, doubtless, been suggested by the complaints which the commissioners have made in their successive annual reports respecting the management of Guy's, St. Luke's, and Bethlehem Hospitals.

*Commissioners may make regulations for the government of licensed houses.*—The commissioners, by this section, have a great increase of power delegated to them. It is enacted by section 31, It shall be lawful for the commissioners, with the sanction and approbation of her Majesty's principal Secretary of State, from time to time, to make regulations for the government of any house licensed for the reception of lunatics; and such regulations of the commissioners, or a copy thereof, shall be transmitted by their secretary to the proprietor or resident superintendent of every licensed house to which the same relate, and shall be abided by and observed therein.

*Time at which the annual reports of the Commissioners are to be made to the Lord Chancellor.*—The report required by section 88 of the former Act to be made by the commissioners in the month of June in every year, is by the 32nd section of the new Act required to be made in or before the month of March in every year, and to be made up to the end of the preceding year.

By the 25th section, *Bethlehem to be under the supervision of the Commissioners in Lunacy.*

To conclude, the following is a general and brief summary of the alterations and amendments contained in the sections of the Act above referred to.

I.

Two or more houses may be included in one licence, if the land be in the same occupation; or if such buildings be separated by a road.

II.

The form of the order of reception is slightly altered. The forms of medical certificates are also changed, requiring the facts to be specified which the medical practitioners signing the certificate have observed, which are to be distinguished from those communicated to them by other persons. (Schedules A and B.)

III.

The same form of notice of admission to the commissioners, upon a patient being admitted into an asylum, is now prescribed for both private and pauper patients. (Schedule C.)

IV.

The orders of reception and medical certificates may, in cases of informality, after the reception of a patient into an asylum, be returned for amendment. (Sect. XI.)

V.

A discharged patient may, with the assent of the commissioners or visitors, be retained in a licensed house, and a relative or friend may, with the like assent, be received therein. (Sect. VI.)

VI.

Patients may, under peculiar and specified circumstances, be admitted into licensed houses upon *one* medical certificate; but *two* additional ones must be obtained within two days after the reception of such patient. The former Act required only *one* additional certificate.

VII.

Single patients in unlicensed houses are placed under the same protections as persons in licensed houses. (Sects. VIII., XIV., XV., XVI., XVII., XVIII.)

VIII.

On recovery of a patient, notice to be given by the superintendent or proprietor to the friends, and, in the case of a pauper, to the guardians, and in default of the patient being discharged or removed within fourteen days after such intimation, notice is to be transmitted to the commissioners or visitors. (Sect. XIX.)

IX.

Notice in case of death is to be sent to the coroner of the county or borough, with a statement setting forth the duration of the disease and cause of death; who may thereupon, if there be any reasonable suspicion, cause an inquest to be held. The particulars transmitted to the coroner to be entered in the Case Book. (Sect. XIX.)

X.

Notice of dismissal for misconduct of nurses or attendants to be sent to the commissioners within one week after the date of such dismissal. (Sect. XXVI.)

XI.

The duties of the commissioners materially facilitated by vesting the powers of the private committee in the person of one of two of the commissioners (sect. 27) by the like provision for the visitation or workhouses (sect. 28), and by their having the legal power to employ persons in special cases to make inquiries and report thereon to them. (Sect. XXIX.)

XII.

Commissioners authorized with the sanction and approbation of one of her Majesty's principal Secretaries of State to make regulations for the government of any licensed house. (Sect. XXXI.)

Such are the principal alterations in the new Act, which now demand the attention of proprietors and medical superintendents; and by referring to the amended schedules, it will be observed that, as far as new private lunacy books and forms are concerned, the following is the state of matters:—The "Visitors' Book," the "Patients' Book," the "Case Book," the "Registry of Admissions," the "Registry of Discharges and Deaths," are none of them altered. The same books which are now provided may continue in use under the new Act. The form of the "Medical Visitation Book," or "Medical Journal and Weekly Report," is however altered. A separate column is appropriated to restraints and seclusions. Under the head of "patients under medical treatment," their bodily disorder is to be specified; and the column is omitted which reported the "state of health of patients and condition of the house or hospital." It will be necessary, therefore, to provide a new "Medical Visitation Book." With respect to the circular notices, the notice of discharge of patients remains the same; but the "order for reception of private and pauper patients, and the notice of admission, and notice of death and cause of death," having their forms altered, new ones must be provided, which will be required by the first of November next.

In the present article we have only set forth the new points in the Lunacy Care and Treatment Bill, many of the provisions of which were obviously required; and we have no doubt will be found to work well. Others, however, appear to us in a doubtful light, but we will not prejudge the effects of their operation. The "Lunatic Asylums Bill," and the "Lunacy Regulation Bill"—one of which chiefly relates to the management of county asylums, and the other to the law relating to commissioners *de lunatico inquirendo* and the management of the property of lunatics—we shall analyse and consider at a future period.

---

